# Comparison of Four Methods for Measuring Heterophoria and Accommodative Convergence over Accommodation Ratio

**DOI:** 10.3390/vision8040062

**Published:** 2024-10-18

**Authors:** Noelia Nores-Palmas, Veronica Noya-Padin, Eva Yebra-Pimentel, Maria Jesus Giraldez, Hugo Pena-Verdeal

**Affiliations:** 1 Applied Physics Department (Optometry Area), Facultade de Óptica e Optometría, Universidade de Santiago de Compostela, 15705 Santiago de Compostela, Spainmjesus.giraldez@usc.es (M.J.G.); 2 Optometry Group, Instituto de Investigación Sanitaria Santiago de Compostela (IDIS), 15706 Santiago de Compostela, Spain

**Keywords:** heterophoria, AC/A ratio, OptoTab SERIES, cover test, Modified Thorington test, Von Graefe

## Abstract

The study aimed to assess the agreement between OptoTab SERIES, alternating Cover Test, Modified Thorington test, and Von Graefe method in measuring heterophoria and accommodative convergence over accommodation (AC/A) ratio. In an initial step, heterophoria was assessed at both distance and near in a cohort of 76 healthy young volunteers using the previously described tests. Subsequently, to determine the AC/A ratio, near-vision measurements were repeated with +1.00 D and −1.00 D lenses. All tests were performed in a randomized order across participants under consistent conditions. Significant differences were found between the Modified Thorington test and all other tests at distance (Wilcoxon test, all *p* ≤ 0.001) and between Von Graefe and all other tests at near (Wilcoxon test, all *p* ≤ 0.005). Regarding the AC/A ratio, significant differences were observed between all methods in +1.00 D AC/A ratio, except for the Modified Thorington test vs. the alternating Cover Test (Wilcoxon test, *p* = 0.024). In the −1.00 D AC/A ratio, differences were observed between OptoTab POCKET and all the other tests (Wilcoxon test, all *p* ≤ 0.001). The results indicate that all methods are interchangeable except the Modified Thorington test at distance and Von Graefe at near. For the AC/A ratio, only the Modified Thorington test is interchangeable with the alternating Cover Test using +1.00 D lenses and all are interchangeable using −1.00 D lenses except OptoTab POCKET.

## 1. Introduction

Accommodative and vergence anomalies are a common group of visual dysfunctions that reduce the efficiency of the visual system [1]. In an optometric regular visual assessment, measuring the heterophoria is important to assess the presence of vergence visual dysfunction and to support the diagnosis of non-strabismic binocular vision anomalies that are very common in a pre-presbyope population such as vergence insufficiencies or excesses or basic deviation [2]. Heterophoria measurement is a useful indicator not only in diagnosis but also in the choice for treatment of certain binocular disorders [3,4,5]. There are already some studies that discuss the relationship between binocular vision disorders and learning or reading disabilities. This relationship is not clear yet, but it is known that, for example, children suffering from hyperactivity disorder have higher scores on convergence insufficiency symptomatology questionnaires [6,7]. Moreover, an absence of a proper diagnosis due to the reliance on unreliable tests may consequences such as amblyopia or loss of binocular function and stereopsis [8,9].

A heterophoria has been defined as a latent deviation of the visual axis that manifests itself in the absence of stimuli, in contrast to strabismus which has been defined as a manifest deviation [10,11]. These can be horizontal (divided in eso- and exophoria) vertical (divided in hyper- and hypophoria), or torsional (incyclo- and excyclophoria) [12]. Its measurement depends on tonic and proximal convergence, accommodative response, vergence adaptation, and gaze position. Heterophoria values are given in prismatic dioptres [13,14]. In addition, the literature has assumed that accommodation is usually primarily driven by blur whereas vergence is driven primarily by disparity, nevertheless, both parameters have an inherent influence on each other by a neural cross-link between them [5,15,16]. The main clinical parameter to measure this influence of one over the other is the ratio of the accommodative convergence (AC) over accommodation (A) which indicates the relationship between the amount of convergence produced by a stimulus to accommodate and the amount of accommodation that produces that convergence [17]. The measurement of the accommodative convergence over accommodation (AC/A) ratio is used clinically to determine the etiology and classification of certain binocular disorders [18].

In clinical practice, several classical methods are utilized to determine the heterophoria magnitude. These methods include the alternating Cover Test, Maddox rod test, Von Graefe method, Modified Thorington test, and Howell Card [3,5,19]. The alternating cover test is considered to be the reference method for distance assessment, while the Modified Thorington test has shown the best repeatability for near assessment [20]. Additionally, there are newly proposed technologies based on a digital tablet or polarized screen with compatible filter glasses to measure heterophorias, such as the OptoTab SERIES, that have not yet undergone reliability testing. OptoTab SERIES (www.smarthings4vision.es, accesed on 17 October 2024), is composed of OptoTab POLAR and OptoTab POCKET. OptoTab POLAR is a 24″ polarized screen used for distance measurements with polarized accessories for those tests that require dissociation between eyes, such as heterophoria measurement. On the other side, OptoTab POCKET is an 11″ anaglyph screen used for near measurement, with red–green glasses to dissociate the eyes in those tests where it is required. These devices, with tests that allow not only heterophoria but also other aspects of binocular function to be assessed quickly, using intuitive and easy-to-understand tests, are valuable tools for reducing the overall time needed for visual checks. The interchangeability of the different tests has been assessed by previous studies. It was found that these methods are not interchangeable, possibly due to differences in dissociation methods or accommodation control [3,4,10,12,14,19,21,22]. Furthermore, various factors, including stimulus, environmental illumination, quantification method, patient status, objectivity, and examiner skills, can affect the measurements and contribute to errors [23]. Therefore, the present study aims to compare four methods (OptoTab SERIES, alternating Cover Test, Modified Thorington test, and Von Graefe method) to assess their agreement for measuring distance and near heterophoria and AC/A ratio.

## 2. Materials and Methods

### 2.1. Participants

A total of 100 volunteer participants (33 men and 67 women) with a mean age of 22.7 ± 2.8 years in a range from 18 to 34 were recruited among university students. Participants were initially required to complete a questionnaire to record age, biological sex, and ocular and/or systemic disease history. Subsequently, an optometrist conducted the subjective refraction using the fogging technique [24]. The resulting correction was then used to measure the Best Corrected Visual Acuity (BCVA) and binocular vision parameters of each participant. Based on the recorded data, the inclusion and exclusion criteria for being finally included in the study were as follows [10,20,21,25,26,27]:

- A BCVA greater or equal to 1.0 Snellen decimal in each eye at distance and near.
- No accommodative or vergence anomalies; cut-off values are provided in Table 1.
- No vertical or cyclovertical phorias.
- No history of a previous or active ocular pathology, infection, dry eye disease, meibomian gland dysfunction, refractive surgery, strabismus, nystagmus, or amblyopia.
- No medication or disease that could affect accommodation, fusional vergences, or ocular motility.

Informed consent was obtained from all volunteer participants to be included in the study. All procedures were in accordance with the ethical standards of the responsible committee on human experimentation (institutional and national) and with the Helsinki Declaration and its later amendments.

### 2.2. Test Procedures

Once included in the study, a battery of four methods for the measurement of heterophoria was performed on each participant, where only the horizontal deviations were considered. All tests were completed in a single visit and performed in random order on each participant to avoid practice and fatigue from influencing results. Heterophoria was measured at a distance (6 m), except for the Modified Thorington test (3 m), and near (40 cm) using four different methods: OptoTab SERIES, alternating Cover Test, Modified Thorington test, and Von Graefe. All tests were performed under the same conditions, comprising constant illumination, with all overall lights on and supplemented by a standup light to illuminate the optotypes used at near vision, assessment at the primary viewing point, and participants with their best refractive correction (if any) throughout the course of the tests [10]. After measuring heterophorias, the procedure was repeated with both +1.00 D and −1.00 D lenses mounted in a flipper held by the participant in front of the glass to calculate the AC/A ratio. The difference between heterophoria measurement with and without the +1.00 D and −1.00 D lenses was the AC/A ratio value. In the case of Von Grafe, +1.00 D and −1.00 D lenses were allocated in the phoropter.

#### 2.2.1. OptoTab SERIES

**Figure 1:**
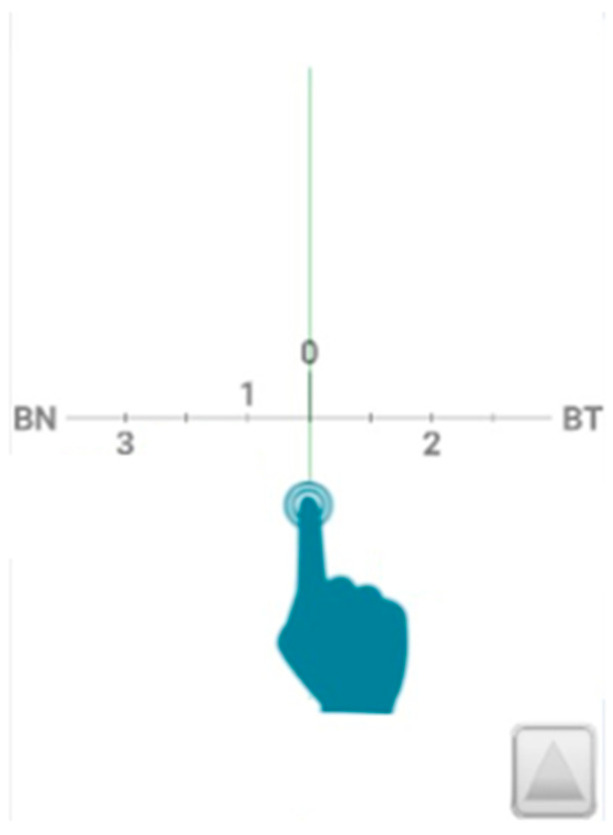
OptoTab measuring example for heterophoria assessment.

A pair of two specifically designed video terminals from OptoTab SERIES was used to measure the horizontal heterophoria magnitude: OptoTab POLAR 24″ SMT4V screen (Smarthings4vision, Zaragoza, Spain) for distance and OptoTab POCKET FHD 10″ tablet (Smarthings4vision, Spain) for near testing. Both terminals were based on the graphical display of the Modified Thorington test for the heterophoria assessment [10,20,21]. A pair of polarized glasses were used in distance measurements while a pair of red/green glasses were used for near testing. With both polarized and anaglyph glasses, while one eye saw the scale, the other eye saw the line. The point where the line crossed the scale represented the heterophoria value. The participants were instructed to indicate in which number and direction of the scale the vertical line on the scale was observed. If the line was seen at the right of the center, it would be an esophoria whereas if the line was seen at the left, it was an exophoria. This number (to the nearest 0.5∆) was the heterophoria value recorded. Figure 1 shows graphically the measurement technique.

#### 2.2.2. Cover Test

For distance testing, the fixation stimulus was a row of letters on a screen of 0.8 VA Snellen and participants were asked to maintain fixation on one of the letters. For near testing, a stick with a row of letters of 0.8 VA Snellen held by the participant was used. Participants were asked to look at the isolated letter on the top of the stick while the examiner measured the heterophoria. The unilateral cover test was always the first test, followed by the alternating cover test. The unilateral cover test was performed to check there was no strabismus. The standard-sized cover paddle was placed on the participant’s right eye for about 2 s, after which it was removed. Both eyes were exposed for 2–3 s, and the opaque occluder was placed on the opposite eye for 2 s. Each time one eye was occluded, examiners observed the unoccluded eye for a fixation movement: an inward movement indicated exotropia and an outward movement indicated esotropia [22].

For the alternating cover test, the participant held the near fixation stick. The same standard-sized cover paddle was used to occlude first the right eye and then the left eye for a minimum of 5 s to minimize the effect of vergence adaptation [19]. A prism bar with powers of 1, 2, 4 to 20∆ in 2∆ steps was placed no further than 1 cm from the right eye to objectively obtain the first neutral endpoint by adding prisms until no movement could be seen [20]. An inward movement indicates exophoria and requires base-in prisms to neutralize, while an outward movement indicates esophoria and requires base-out prisms to neutralize.

#### 2.2.3. Modified Thorington Test

A pair of two specifically designed Phoria Cards (Promoción Optométrica, Burgos, Spain) were used to measure heterophorias with the Modified Thorington test [10,20,21]. The cards were calibrated for distance testing at 3 m and 40 cm for near testing. A Maddox rod streak was placed ahead of the participant’s right eye in a horizontal position. The participants were instructed to look at the zero in the center of the card and to keep it clear. Examiners used a punctual light in the center hole of the card and asked participants which number was closest to the vertical line [10]. If the line was seen at the right of the center, it was an esophoria meanwhile if the line was seen at the left, it was an exophoria. This number (to the nearest 0.5∆) was the heterophoria value recorded [10,20].

#### 2.2.4. Von Graefe

A 0.5 to 0.8 VA column of letters was used as the stimulus for heterophoria measurements at distance while a 0.8 VA column of letters on a Snellen Chart was used for measurements at near. A phoropter with a base-in prism of 12∆ ahead of the right eye and a base-up of 6∆ ahead of the left eye of the participant was used to measure the horizontal heterophoria. Participants were instructed to keep the letters of the image below focused and to notify when two columns are lined up. The number of the prism placed ahead of the right eye of the participant was the heterophoria value at this point. In the cases where the exophoria was more than 12∆ the dissociating prism was increased. Similarly to other studies and to avoid biased results because of the possible lack of understanding by the patient, this procedure was repeated three times at both distance and near; the final values were computed as the mean of the three measurements performed [19,28].

### 2.3. Statistical Analysis

SPSS statistical software v.25.0 for Windows (SPSS Inc., Chicago, IL, USA) was used for data analyses. Significance was set at a *p* ≤ 0.05 for all the analyses. Before analysis, the normal distribution of the data for heterophoria measurements at distance and near on each procedure was checked using the Kolmogorov–Smirnov test; all parameters showed a non-normal distribution (Kolmogorov–Smirnov, all *p* < 0.05); hence, non-parametric tests were used [29]. Differences between all methods were assessed using the Friedman test, whereas the Wilcoxon test was used to detect significant pairwise differences; to avoid type I errors arising from multiple comparisons in the heterophoria analysis between methods, statistical significance for the Wilcoxon test was divided by the number of comparisons performed to give a *p* ≤ 0.005. Correlation between variables was described as weak (0.20–0.40), moderate (0.41–0.60), good (0.61–0.80), or strong (0.81–1.00).

## 3. Results

Table 2 shows the descriptive statistics of the results obtained in the battery of tests performed as inclusion criteria for the study. For PFV and NFV three values are indicated: blur/break/recovery. From the initial sample, a total of 24 participants were excluded for not meeting the inclusion criteria: VA lower than 1.0 (6 participants), previous diagnosis of amblyopia (1 participant), strabismus (3 participants), near point of convergence (NPC) larger 10 cm (6 participants), decrease accommodation amplitude (AA) (3 participants), or low vergences capacities (4 participants) and a combination of strabismus and amblyopia (1 participant).

Considering all tests, heterophoria values at distance varied between −14.00 Δ and 9.00 Δ and between −30.00 Δ and 15.00 Δ at near.

### 3.1. Differences and Correlations Between Heterophoria Measurements at Distance

Table 3 provides descriptive statistics for the four measuring heterophoria methods at distance. Those values with negative signs are for exophorias and those others with positive signs refer to esophoria [30]. A statistical difference was obtained between the four methods (Friedman test; *p* < 0.001, Table 3). When the differences between paired methods for distance vision were assessed, only the Modified Thorington test showed significant differences from all the other tests (Wilcoxon Test; all *p* < 0.001). Comparing alternating Cover Test vs. OptoTab POLAR, vs. Von Graefe first or vs. Von Graefe average measurement, Von Graefe first measurement vs. OptoTab POLAR, Von Graefe average measurement vs OptoTab POLAR, and between Von Graefe first and average measurement no significant differences were found (Wilcoxon Test; all *p* ≥ 0.052).

Table 4 shows the correlation between the four methods for measuring heterophoria at distance. There was a weak positive correlation between OptoTab POLAR and Modified Thorington test, OptoTab POLAR and Von Graefe first measurement, and OptoTab POLAR and Von Graefe average measurement (Spearman Rho test; all r ≥ 0.305, all *p* ≤ 0.007) and a moderated positive correlation between alternating Cover Test and OptoTab POLAR, alternating Cover Test and Modified Thorington test, alternating Cover Test and Von Graefe first measurement, alternating Cover Test and Von Graefe average measurement, Modified Thorington test and Von Graefe first measurement, and Modified Thorington test and Von Graefe average measurement (Spearman Rho test; all r ≥ 0.410, all *p* ≤ 0.001). A strong correlation was found between the first and the average Von Graefe measurement (Spearman Rho test; r = 0.973, *p* < 0.001).

### 3.2. Differences and Correlations Between Heterophoria Measurements at Near

Table 3 provides descriptive statistics for the four measuring heterophoria methods at near vision. There was a statistical difference in the measurements of heterophoria at near (Friedman test; *p* = 0.020, Table 3). When the difference between paired methods for near vision was assessed, the Von Graefe measurements were the only results that showed significant differences, both considering the first measure and the mean of the three measures (Wilcoxon Test; all *p* ≤ 0.005). All the other methods showed no significant differences (Wilcoxon Test; all *p* ≥ 0.293).

Table 4 shows the correlation between the four methods for measuring heterophoria at near vision. There was a moderated positive correlation between alternating Cover Test and OptoTab POCKET, Modified Thorington test and OptoTab POCKET, OptoTab POCKET, and the Von Graefe first measurement and average measurement (Spearman Rho test; all r ≥ 0.454, all *p* < 0.001) and a good positive correlation between the alternating Cover Test and the Von Graefe first measurement and average measurement and between the Modified Thorington test and the Von Graefe first and average measurement (Spearman Rho test; all r > 0.720, all *p* < 0.001). A strong correlation was found between the alternating Cover Test and Modified Thorington test and the two Von Graefe measurements (Spearman Rho test; both r ≥ 0.857, *p* ≤ 0.001)

### 3.3. Differences and Correlations Between AC/A Measurements

Table 3 provides descriptive statistics for the four AC/A measuring methods with +1.00 and −1.00 D lenses. There were significant differences between the four methods using both +1.00 and −1.00 D lenses (Friedman test; *p* < 0.001). When the difference between paired methods with +1.00 D lenses was assessed, all methods showed a significant difference (Wilcoxon test; all *p* ≤ 0.001), except for the Modified Thorington test vs. alternating Cover Test (Wilcoxon test; *p* = 0.024). For the −1.00 D measurements, three methods showed statistical differences (OptoTab POCKET vs. alternating Cover Test; the Modified Thorington test vs. OptoTab POCKET and Von Graefe vs. OptoTab POCKET; Wilcoxon test; all *p* ≤ 0.001) while no statistical differences were found between the Modified Thorington test vs. alternating Cover Test, Von Graefe vs. the Modified Thorington test, and Von Graefe vs. alternating Cover Test (Wilcoxon test; all *p* ≥ 0.025).

Table 4 shows the correlation between the four AC/A methods measuring with +1.00 and −1.00 D lenses. With +1.00 D lenses, correlation only was found between the alternating Cover Test and Von Graefe (Spearman Rho test; r = 0.364, *p* = 0.001). With −1.00 D lenses, a weak positive correlation was found between alternating Cover Test and OptoTab POCKET, alternating Cover Test and Von Graefe, and OptoTab POCKET and Modified Thorington test (Spearman Rho test; all r ≥ 0.262, all *p* ≤ 0.022).

## 4. Discussion

Heterophoria assessment is a fundamental part of the visual exam in daily clinical practice because of all the information that can be obtained from it. In addition to traditional methods, new technologies are now available for measurement, although their reliability has not been checked yet. The AC/A ratio is used clinically to classify certain types of strabismic and non-strabismic dysfunction and to choose the best treatment for some binocular anomalies [17,31]. In the assessment of heterophoria, it is crucial to distinguish between clinically and statistically significant differences. Clinically significant differences refer to changes that impact patient management and outcomes, typically exceeding the minimum discernible difference of 2Δ established in the literature [3]. Clinically significant differences refer to changes that impact patient management and outcomes, typically exceeding the minimum discernible difference of 2Δ established in the literature [3,10]. For instance, while certain tests may yield statistically significant results, they may not reflect meaningful variations in clinical practice. Studies have shown that methods such as the Modified Thorington test exhibit higher repeatability compared to others, like the von Graefe method, which may produce larger variabilities [3,10]. This variability underscores the importance of considering both intra- and inter-examiner reliability when interpreting results [3,4,20]. Thus, a careful evaluation of measurement techniques is necessary to ensure that observed differences are not merely mathematical artifacts but hold true clinical relevance.

During the present study in the distance vision heterophoria assessment, significant differences were obtained between the Modified Thorington test and all the rest of the tests, while in near vision significant differences were only obtained between Von Graefe and all the other tests. These results can be interpreted differently considering that for some tests the minimum discernible difference by the examiner is 2Δ. Consequently, while the differences observed in distance vision may be statistically significant between tests, they may not have clinical relevance. The findings are consistent with previous studies in terms of distance [3,21] or near-vision heterophoria [3,12,19,21]. Similar to the present study, Sanker et al. [10] found significant differences between the Modified Thorington test and Von Graefe; however, contrary to the present study, those authors found significant differences between the alternating Cover Test and the Modified Thorington test. Previous studies have concluded that the methods are not interchangeable, neither in distance nor in near vision [3,10,12,19,21,22]. Differences between tests may be explained because of the different dissociation methods, stimuli, quantification methods, patient conditions, and examiner skills. In the present study, the dissociation methods differed between tests. In the Cover Test, the fusion is completely broken with an opaque occluder. The Von Graefe method induces dissociation by placing a vertical prism in front of one eye and a horizontal prism in front of the other, resulting in the perception of two separate images. The OptoTab achieves dissociation using red–green glasses for near vision and polarised glasses for distance vision. Finally, Thorington employs a Maddox filter ahead of the right eye [10,19]. Moreover, participants seem to experience more difficulty with anaglyph-type glasses (e.g., OptoTab SERIES near vision heterophoria) than polarized (e.g., OptoTab SERIES distance vision heterophoria); the use of red/green targets appears to create an obstacle to fusion, particularly for patients with moderate to severe suppression. Previous studies [32,33,34] suggested that those difficulties may be generated by the currently available glasses which can induce significant inequalities in retinal illuminance that exacerbate suppression tendencies as well as “ghost images” and lateral chromatic aberration that could affect binocular vision; however, the red/green glasses provided here are specifically designed for the use with the OptoTab SERIES near’s device. Concerning the stimulus size, it is known that accommodation affects heterophorias and vice versa [35], therefore the accommodation control should be an important factor during measurement; in this study, alternating Cover Test and Von Graefe measured heterophorias using a similar accommodative stimulus, while the Modified Thorington test and OptoTab SERIES used a non-accommodative stimulus. Moreover, some authors state that minimal ocular deviation visible using alternating Cover Test, the gold standard for assessing distance vision, is 2Δ. This may introduce variability, as other tests can measure from 0Δ [36,37]. Differences on the scale of measurement between tests may be also a source of error. While with alternating Cover Test primarily allows for quantification steps of 2Δ, the Von Graefe method had steps of 1Δ, and both Modified Thorington test and OptoTab SERIES had 0.5Δ steps. This may lead to mathematically significant differences, but they are unlikely to be clinically significant in practice. On the other hand, measurements using a phoropter can be influenced by proximal vergence, head and eyes position, and reduced peripheral visual field [20,21]. Some authors also have pointed out that high values of heterophoria may have an impact on the discrepancies between test results [19]. All those differences in the measurement principles could be the origin of those discrepancies between tests.

Concerning the AC/A ratio, according to the present study results, none of the methods are interchangeable with +1.00 D lenses, except for the Modified Thorington test and alternating Cover Test. However, with −1.00 D lenses, only OptoTab POCKET showed statistical differences with the other methods. A previous study showed higher variability in Von Graefe values compared to the Modified Thorington test; this study has not discussed the differences between the two methods [38]. Other previous studies compared different methods to calculate the AC/A ratio (e.g., heterophoria method vs. calculated method) but did not compare the same AC/A ratio with different devices; therefore, results should be interpreted with caution [17,31]. The literature has assumed that accommodation is driven primarily by blur while vergence is driven primarily by disparity; however, both have an influence on the other by the neural cross-links between the two systems (vergence–accommodation and accommodative–vergence, respectively) [5,6,8,9,15,16]; a decrease in the capacities in one component of this system may influence the efficiency of the other to compensate the deficiency. Similar to the heterophoria assessment, the discrepancy in results obtained in the present study could be influenced by this close relationship since some of the studied methods are based on accommodative stimuli while others are based on non-accommodative stimuli, which would be in line with the results obtained by Satou et al. [31], which found significant differences between methods depending on whether the stimulus was accommodative or non-accommodative. Miyata et al. [39] found that AC/A ratio measurements are biased by the patient accommodation lag. It is known that myopes have a higher accommodation lag than emmetropes and hyperopes, hence refraction could have been a source of error in this study [40].

The main strength of the present study is the devices employed since new assessment technology methods (OptoTab SERIES) had been compared to “classical methods” (alternating Cover Test, Von Graefe, Modified Thorington test, etc.) both for the heterophoria and AC/A ratio. In addition, the sample size was larger, and inclusion criteria were stricter compared with other previous studies [4,10,12]. The main limitation of the present study was the sample characteristics since only young healthy participants were included, regardless of their prescription and whether they wore glasses or contact lenses; future studies should include people who suffered some ocular vergence dysfunction to find out whether methods are interchangeable in all situations. Another relevant limitation of the present study was that each measurement was taken only once, rather than averaging multiple measurements. This approach was chosen to align with typical clinical practice; however, it may introduce variability, particularly in cases where participant fatigue could affect the results. Future studies should consider taking multiple measurements to reduce variability and improve the robustness of the findings.

## 5. Conclusions

In conclusion, for a healthy young population, all methods are interchangeable for heterophoria measurements at distance except the Modified Thorington test, with which more esophoric values were obtained, and all of them are interchangeable at near measurements except Von Graefe, with which more exophoric values were obtained. In terms of AC/A ratio measurements, all are interchangeable using −1.00 D lenses except OptoTab and none of them are interchangeable with +1.00 D lenses except the Modified Thorington test and alternating Cover Test. It is essential to investigate whether these results can be applied to populations with binocular vision disorders.

## Figures and Tables

**Table 1:** Cut-off values for the initial screening test to discard the possible presence of accommodative or binocular anomalies [1,2]. VA: Visual acuity; AA: Amplitude of accommodation. NPC: Near point of convergence. PFV: positive fusional vergences; NFV: negative fusional vergences.

Test	Method	Cut-Off for Inclusion	
Monocular VA	Snellen	≥1.0	
AA	Push-up test (single 0.8 letter)	≥18–1/3 age	
NPC	Push-up test (single 0.8 letter)	Breakpoint < 10 cm (Outer canthus reference point for the distance)	
Deviation at distance (6 m) and near (40 cm)	Unilateral Cover Test	No tropia	
Soft vergences	Phoropter diasporameter	Distance vision (6 m)	PFV ≥ 6/5Δ
			NFV ≥ 4/2Δ
		Near vision (40 cm)	PFV ≥ 10/7Δ
			NFV ≥ 7/5Δ
Near stereopsis	Random dot test at 40 cm	<70 s of arc	

**Table 2:** Descriptive statistics for sample (n = 76) meeting inclusion criteria. VA: Visual acuity; OD: Ocular dexter. OS: Ocular sinister; AA: Accommodation amplitude; NPC: Near point of convergence; PFV: Positive fusional vergences; NFV: Negative fusional vergences; SD: standard deviation; IQR: interquartile range.

Test			Descriptive Statistics			
			Mean	Median	SD	IQR
VA (Decimal)	OD		0.99	0.98	0.04	0.98–1.00
	OS		0.99	0.98	0.02	0.98–1.00
AA (D)	OD		11.29	11.11	1.71	10.00–12.50
	OS		11.32	11.11	2.21	10.00–12.38
NPC (cm)	Break		1.20	0.00	2.60	0.00–0.00
	Recovery		1.44	0.00	3.20	0.00–0.00
Soft vergences (Δ)	Distance vision (6 m)	PFV	15.82/21.38/12.05	14.50/22.00/12.00	6.94/7.31/5.83	10.25–20.00/18.00–26.00/8.00–15.00
		NFV	8.05/8.61/5.53	8.00/8.00/6.00	2.60/2.55/1.90	6.00–10.00/6.00–10.00/4.00–7.00
	Near vision (40 cm)	PFV	26.47/27.64/15.20	28.00/28.00/14.00	7.12/7.21/7.73	21.00–32.00/22.00–33.50/9.00–20.00
		NFV	15.29/17.38/11.87	16.00/18.00/12.00	5.56/5.72/5.59	12.00–18.00/14.00–22.00/7.25–16.00
Near stereopsis (seconds of arc)			30.79	25.00	11.96	20.00–40.00

**Table 3:** Descriptive statistics and analysis of differences (Friedman) for four measuring methods at distance and near vision and AC/A ratio. n = 76. IQR: Interquartile Range.

	Method	Median	IQR	*p*
Distance vision	OptoTab POLAR [∆]	0.0	0.0–0.0	<0.001
	Alternating Cover Test [∆]	0.0	0.0–0.0	
	Modified Thorington test [∆]	0.5	0.0–2.4	
	Von Graefe first measurement [∆]	0.0	−2.0–2.0	
	Von Graefe average measurement [∆]	0.2	−2.0–2.0	
Near vision	OptoTab POCKET [∆]	0.0	−0.5–0.0	0.020
	Alternating Cover Test [∆]	−0.3	−3.8–2.0	
	Modified Thorington test [∆]	0.0	−3.0–2.0	
	Von Graefe first measurement [∆]	−1.5	−7.8–2.0	
	Von Graefe average measurement [∆]	−1.7	−6.9–2.0	
+1.00 D AC/A ratio	OptoTab POCKET [∆]	0.5	0.0–1.0	<0.001
	Alternating Cover Test [∆]	2.0	1.0–4.0	
	Modified Thorington test [∆]	2.0	1.0–3.0	
	Von Graefe [∆]	3.7	2.0–5.7	
−1.00 D AC/A ratio	OptoTab POCKET [∆]	0.5	0.0–1.4	<0.001
	Alternating Cover Test [∆]	2.0	1.0–4.8	
	Modified Thorington test [∆]	2.0	1.0–4.0	
	Von Graefe [∆]	3.7	2.0–5.7	

**Table 4:** Correlation (Spearman Rho test) between the four methods for measuring heterophoria at distance and near vision and AC/A ratio.

		Alternating Cover Test [∆]	Modified Thorington Test [∆]	Von Graefe First Measurement [∆]	Von Graefe Average Measurement [∆]
Distance vision	OptoTab POLAR [∆]	r = 0.410 *p* < 0.001	r = 0.367 *p* = 0.001	r = 0.305 *p* = 0.007	r = 0.337 *p* = 0.003
	Alternating Cover Test [∆]		r = 0.474 *p* < 0.001	r = 0.493 *p* < 0.001	r = 0.541 *p* < 0.001
	Modified Thorington test [∆]			r = 0.414 *p* < 0.001	r = 0.434 *p* < 0.001
	Von Graefe first measurement [∆]				r = 0.973 *p* < 0.001
Near vision	OptoTab POCKET [∆]	r = 0.454 *p* < 0.001	r = 0.532 *p* < 0.001	r = 0.518 *p* < 0.001	r = 0.539 *p* < 0.001
	Alternating Cover Test [∆]		r = 0.857 *p* < 0.001	r = 0.769 *p* < 0.001	r = 0.774 *p* < 0.001
	Modified Thorington test [∆]			r = 0.720 *p* < 0.001	r = 0.726 *p* < 0.001
	Von Graefe first measurement [∆]				r = 0.984 *p* < 0.001
+1.00 D	OptoTab POCKET [∆]	r = 0.139 *p* = 0.231	r = 0.161 *p* = 0.165	r = −0.084 *p* = 0.470	
	Alternating Cover Test [∆]		r = 0.105 *p* = 0.366	r = 0.364 *p* = 0.001	
	Modified Thorington test [∆]			r = 0.101 *p* = 0.384	
−1.00 D	OptoTab POCKET [∆]	r = 0.359 *p* = 0.001	r = 0.335 *p* = 0.003	r = 0.170 *p* = 0.143	
	Alternating Cover Test [∆]		r = 0.315 *p* = 0.006	r = 0.262 *p* = 0.022	
	Modified Thorington test [∆]			r = 0.119 *p* = 0.304	

## Data Availability

The data are unavailable due to privacy restrictions.
